# Co-targeting of Bcl-2 and mTOR pathway triggers synergistic apoptosis in BH3 mimetics resistant acute lymphoblastic leukemia

**DOI:** 10.18632/oncotarget.5156

**Published:** 2015-09-16

**Authors:** Stefano Iacovelli, Maria Rosaria Ricciardi, Matteo Allegretti, Simone Mirabilii, Roberto Licchetta, Paola Bergamo, Cinzia Rinaldo, Ann Zeuner, Robin Foà, Michele Milella, James A. McCubrey, Alberto M. Martelli, Agostino Tafuri

**Affiliations:** ^1^ Department of Cellular Biotechnologies and Hematology, Sapienza University of Rome, Italy; ^2^ Hematology, “Sant'Andrea” Hospital - Sapienza University of Rome, Department of Clinical and Molecular Medicine, Rome, Italy; ^3^ Institute of Molecular Biology and Pathology, National Research Council, Rome, Italy; ^4^ Department of Hematology, Oncology and Molecular Medicine, Istituto Superiore di Sanità, Rome, Italy; ^5^ Department of Medical Oncology, Regina Elena National Cancer Institute, Rome, Italy; ^6^ Department of Microbiology and Immunology, Brody School of Medicine at East Carolina University, Greenville, NC, USA; ^7^ Department of Biomedical and Neuromotor Sciences, University of Bologna, Italy

**Keywords:** acute lymphoblastic leukemia, targeted therapies, BH3 mimetic resistance, mTOR inhibition, Mcl-1

## Abstract

Several chemo-resistance mechanisms including the Bcl-2 protein family overexpression and constitutive activation of the PI3K/Akt/mTOR signaling have been documented in acute lymphoblastic leukemia (ALL), encouraging targeted approaches to circumvent this clinical problem. Here we analyzed the activity of the BH3 mimetic ABT-737 in ALL, exploring the synergistic effects with the mTOR inhibitor CCI-779 on ABT-737 resistant cells. We showed that a low Mcl-1/Bcl-2 plus Bcl-xL protein ratio determined ABT-737 responsiveness. ABT-737 exposure further decreased Mcl-1, inducing apoptosis on sensitive models and primary samples, while not affecting resistant cells. Co-inhibition of Bcl-2 and the mTOR pathway resulted cytotoxic on ABT-737 resistant models, by downregulating mTORC1 activity and Mcl-1 in a proteasome-independent manner. Although Mcl-1 seemed to be critical, ectopic modulation did not correlate with apoptosis changes. Importantly, dual targeting proved effective on ABT-737 resistant samples, showing additive/synergistic effects. Together, our results show the efficacy of BH3 mimetics as single agent in the majority of the ALL samples and demonstrate that resistance to ABT-737 mostly correlated with Mcl-1 overexpression. Co-targeting of the Bcl-2 protein family and mTOR pathway enhanced drug-induced cytotoxicity by suppressing Mcl-1, providing a novel therapeutic approach to overcome BH3 mimetics resistance in ALL.

## INTRODUCTION

Although treatment of pediatric and Philadelphia-positive ALL patients has shown continuous progress, adult patients, especially those characterized by unfavorable prognostic factors, still present high rates of relapse and develop chemo-resistance during the course of the disease, failing to achieve further remissions and ultimately dying [1, 2].

Overexpression of the anti-apoptotic Bcl-2 family proteins is commonly observed in hematological malignancies, including ALL. By blocking apoptosis induction, Bcl-2 allows leukemic cell to survive and proliferate irrespective of death signals that physiologically avoid oncogenesis transformation [3–6]. Anti-apoptotic members perform their activity by binding and sequestering Bax and Bak, thus preventing mitochondria interaction [7, 8]. An imbalanced expression of pro- and anti-apoptotic proteins has been documented in ALL [9] and Bax/Bcl-2 ratio has been inversely correlated to prognosis as it becomes significantly lower at relapse [10]. Moreover, increased expression of Bcl-2 related proteins, frequently observed at diagnosis, is associated with decreased responsiveness to chemotherapy induction and treatment refractoriness [11–14]. Therefore, strategies to inhibit Bcl-2 family proteins have been extensively investigated over the years [15–17] showing high *in vitro* and *in vivo* efficacy.

ABT-737 is a small BH3 mimetic Bcl-2/Bcl-xL inhibitor that has demonstrated impressive pre-clinical activity as single agent against a wide range of solid tumors and hematological malignancies [14, 17–21]. In addition, ABT-737 strongly synergized with chemotherapeutic drugs, supporting its inclusion in the ALL agent *armamentarium* [22, 23]. However, although ABT-737 potently binds to Bcl-2, Bcl-xL and Bcl-w, it weakly interacts with Bfl-1/A1 or Mcl-1 [16, 24]. It has been reported that high expression of Mcl-1 significantly reduced ABT-737 cytotoxicity in several cancers, including ALL [25–28]. Moreover, cells that are initially sensitive to ABT-737 may become resistant by upregulating Mcl-1 at transcriptional or post-translational levels [28, 29]. Consistently, ectopic or pharmacological modulation of Mcl-1 protein levels restored sensitivity to ABT-737 [30, 31], thus indicating Mcl-1 as an important determinant for ABT-737-induced cytotoxicity.

In addition to major cytogenetic alterations, genomic or expression profiling has recently identified the activation of several signal transduction pathways, including phosphoinositide 3-kinase (PI3K)/Akt/mammalian target of rapamycin (mTOR), Ras/Raf/MEK/Erk, Jak/STAT and others as driving features of leukemia [32–34]. mTOR signaling is one of the most frequently dysregulated pathway in ALL and negatively affects patients outcome [35–37]. mTOR is present in two distinct multi-protein complexes, mTOR complex 1 (mTORC1) and mTOR complex 2 (mTORC2) regulating the activity of p70^S6K^ and of the eukaryotic initiation factor (eIF)4E binding protein-1 (4EBP1) and contributing to Akt activation by phosphorylating S473 residue, respectively [38–40]. Among its multiple functions, Akt directly influences the transcription of Bcl-2 family genes as well as other important regulators of apoptosis [36, 41]. Moreover, it has been demonstrated that mTOR is involved in the regulation of Mcl-1 stability [42–44]. Indeed, by activating Akt, mTORC2 inhibits the Gsk-3 dependent phosphorylation of Mcl-1 on S159 residue, blocking its degradation via proteasome [43, 45]. Therefore, inhibition of mTOR activity may disrupts the balance between pro and anti-apoptotic proteins, further enhancing leukemic cell death. In this context, selective blockade of mTOR activity by rapamycin or its derivatives showed to be effective in different hematological neoplasia [46–50]. Notably, CCI-779 (Temsirolimus), RAD001 (Everolimus) and the ATP-competitive mTOR inhibitor INK128 demonstrated anti-leukemic activity on ALL cell lines and primary samples [50–52].

In the present study we analyzed the effects of the BH3 mimetic ABT-737 on ALL cell lines and primary samples, exploring the potential synergistic effects with the mTOR inhibitor CCI-779 to overcome ABT-737 acquired resistance. Moreover, we assessed the functional response of ALL cells to the agent combination prior and after Mcl-1 manipulation to establish its role in mediating ABT-737 resistance.

## RESULTS

### ABT-737 demonstrated anti-leukemic activity on ALL cells

**Figure 1:**
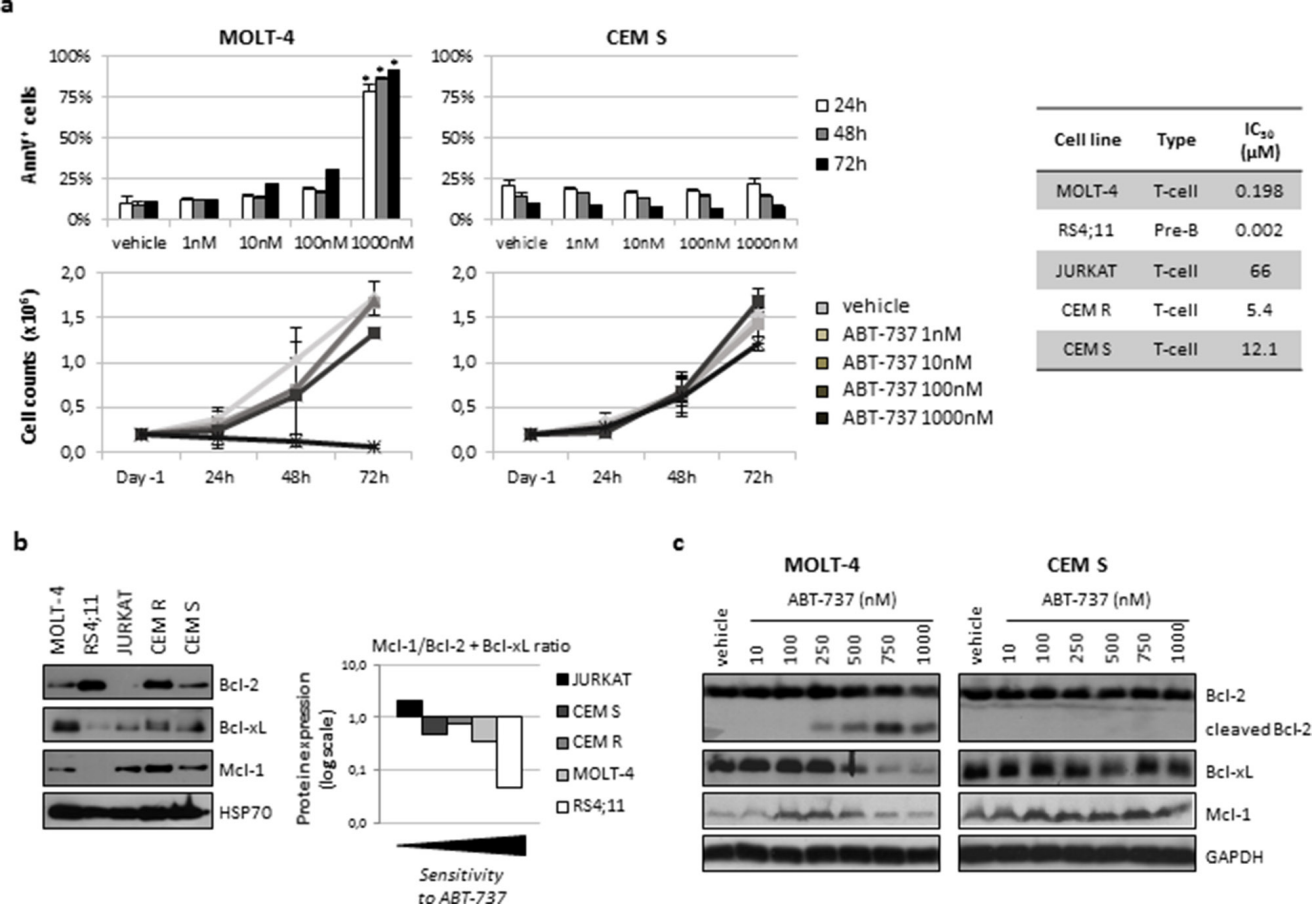
ABT-737 affected cell growth, induced apoptosis and modulated Bcl-2 family protein expression in ALL cell lines **a.** MOLT-4 and CEM S cell lines were exposed to the indicated concentrations of ABT-737 or vehicle (DMSO) up to 72 h. Apoptosis induction (upper panels) and cell counts (lower panels) were assessed by AnnV/PI staining and Trypan blue exclusion method, respectively. Data are shown as mean ± SD of at least three independent experiments. IC_50_s were calculated after 72 h of exposure by using the CalcuSyn software. **b.** Basal expression of selected Bcl-2 family members was analyzed by western blotting in ALL cell lines (left) and quantified by densitometric analysis (right). HSP70 served as loading control. Relative amount of each protein was calculated by dividing the densitometric value of the protein for that of the corresponding HSP70 and then Mcl-1/Bcl-2 plus Bcl-xL ratio was assessed. **c.** Modulation of protein expression following 24 h of exposure to ABT-737 or vehicle (DMSO) in MOLT-4 and CEM S cell lines. Cleavage of Bcl-2 and levels of Bcl-xL and Mcl-1 were assessed by western blotting. GADPH was used as loading control. * for *p* < 0.05.

To recapitulate ABT-737 effects on ALL, we initially assessed ABT-737 cytotoxicity as a single agent on 5 human ALL cell lines, looking for a potential correlation between drug sensitivity and Bcl-2 family members expression. A dose-dependent apoptosis induction was observed at increasing concentrations on the MOLT-4 and RS4;11 cell lines (IC_50_s: 198 and 2 nM, respectively) whereas JURKAT, CEM R and CEM S proved resistant (IC_50_s > 5 μM) (Fig. 1a). Western blot analysis revealed that Bcl-2, Bcl-xL and Mcl-1 were heterogeneously expressed among the cell models (Fig. 1b). Interestingly, resistant cell lines displayed a higher Mcl-1/Bcl-2 plus Bcl-xL protein ratio than sensitive models. Modulation of protein expression induced by ABT-737 treatment was then analyzed in a representative sensitive, MOLT-4, or resistant, CEM S, cell model. ABT-737 exposure caused the cleavage of Bcl-2 and the downregulation of Bcl-xL and Mcl-1 only in the MOLT-4 cell line, whereas their expression resulted not affect in the resistant CEM S model (Fig. 1c).

**Figure 2:**
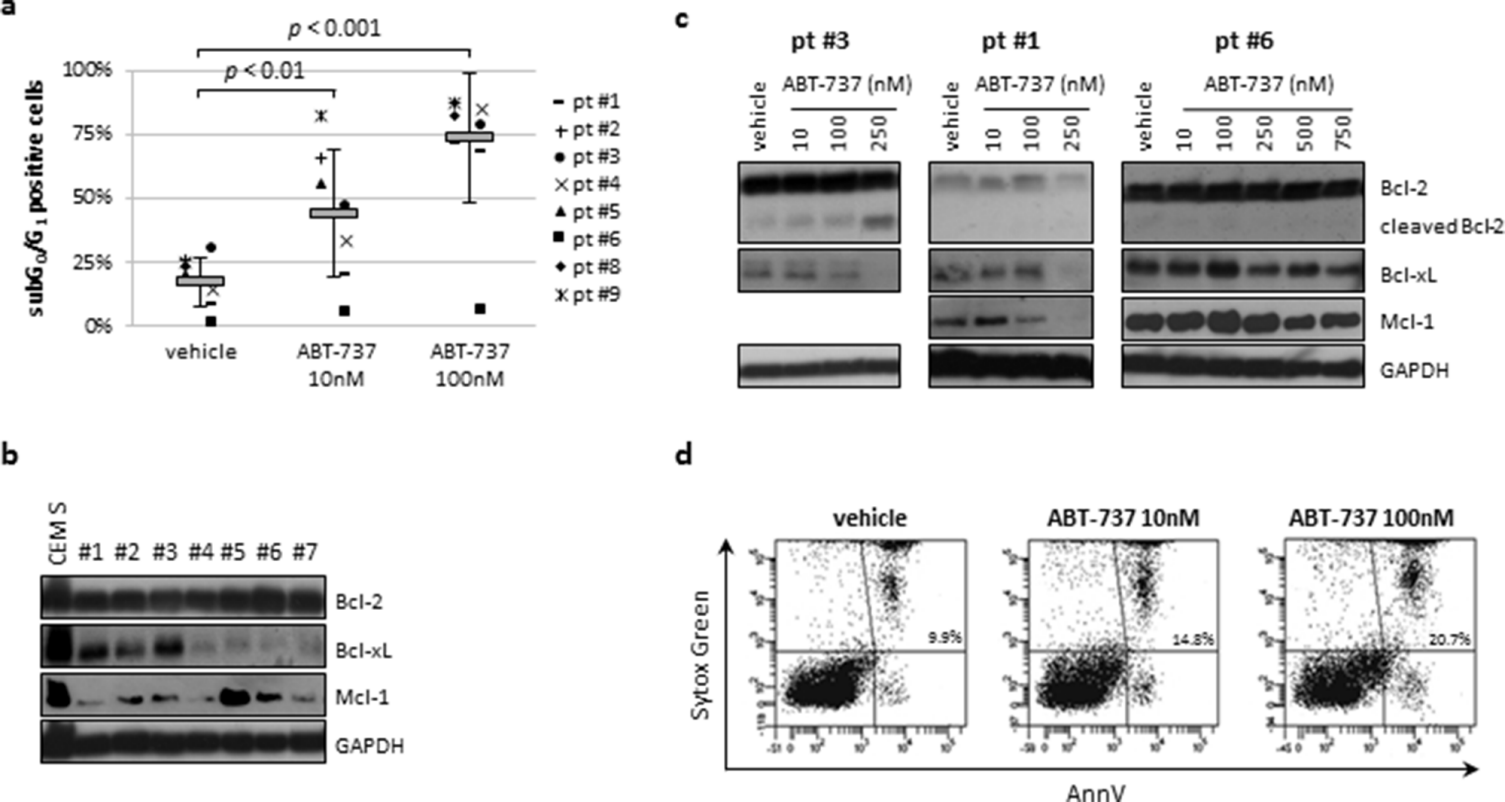
ABT-737 induced apoptosis in ALL primary samples but not in normal CD34^+^ cells **a.** Nine ALL primary samples were treated with ABT-737 or vehicle (DMSO) for 24 h. Apoptosis induction was measured by flow cytometry AO technique and reported as percentage of subG_0_/G_1_ positive cells. **b.** Western blot analysis of basal Bcl-2, Bcl-xL and Mcl-1 protein levels in ALL primary samples with available cell amounts. CEM S cell lines was used as reference. **c.** Modulation of protein expression following 24 h of exposure to ABT-737 or vehicle (DMSO) in three primary samples. Mcl-1 was not assessed in pt #3 due to low amount of protein lysate. GADPH served as loading control. **d.** Representative flow cytometric analysis of ABT-737 exposure on normal CD34^+^ cells. Cytotoxic effects were shown as percentage of AnnV^+^ cells.

We next evaluated the efficacy of ABT-737 on cell growth and apoptosis induction in 9 ALL primary samples. Treatment with increasing concentrations of ABT-737 resulted in a significant decrease of cell counts and induced apoptosis in a dose-dependent manner. Following 24 h of exposure, subG_0_/G_1_ positive cells increased from 17.4 ± 9.3% (vehicle) to 44.2 ± 24.6% (*p* < 0.01) and 73.8 ± 25.3% (*p* < 0.001) at 10 and 100 nM, respectively (Fig. 2a). No statistically significant correlation between ABT-737 sensitivity and clinical features of primary samples (age, WBC) was found. Analysis of Bcl-2, Bcl-xL and Mcl-1 protein levels on 7 evaluable samples showed an elevated expression of Bcl-2 in all primary samples with heterogeneous levels of Bcl-xL and Mcl-1 (Fig. 2a). Of note, pt #5 expressed Mcl-1 at high levels, similar to that of CEM S cell line. According to cell availability, we analyzed protein modulations in 3 primary specimens. Our results demonstrate that ABT-737 exposure caused the appearance of cleaved Bcl-2 and induced the downregulation of Bcl-xL and Mcl-1 in two sensitive ALL samples. Conversely, pt #6 showed unchanged levels of Bcl-2, Bcl-xL and of Mcl-1 (Fig. 2c), proving resistant to ABT-737 (subG_0_/G_1_ cells = 6% at 100 nM). These data overlapped with the expression profiles previously observed on ALL cell lines, supporting the idea that Mcl-1 plays a role in the ABT-737 resistance also in ALL primary samples.

Finally, we examined the effect of ABT-737 on normal CD34^+^ cells isolated from the bone marrow of a healthy volunteer showing no significant increase of apoptosis (Fig. 2d).

### Co-targeting of the Bcl-2 family proteins and of the mTOR pathway exerted synergistic activity on resistant ALL cell lines by downregulating Mcl-1 expression

**Figure 3:**
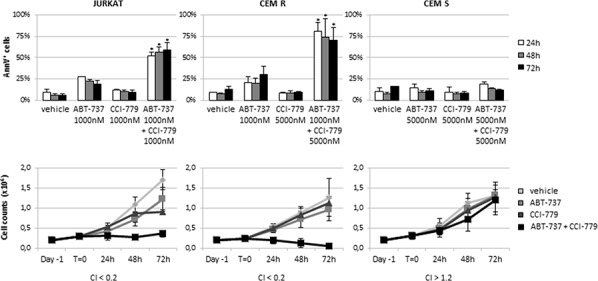
ABT-737/CCI-779 combination triggered synergistic apoptosis in ABT-737 resistant ALL cell lines JURKAT, CEM R and CEM S were exposed up to 72 h to ABT-737 and CCI-779, alone or in combination. Apoptosis induction (upper panels) and cell counts (lower panels) were assessed by AnnV/PI staining and Trypan blue exclusion method, respectively. Results are shown as mean ± SD of at least three independent experiments. CI was calculated by conservative isobologram analysis using the CalcuSyn software. CI values < 0.8 were considered synergistic, between 0.8 and 1.2 additive, > 1.2 antagonistic. * for *p* < 0.01 relative to control conditions.

**Figure 4:**
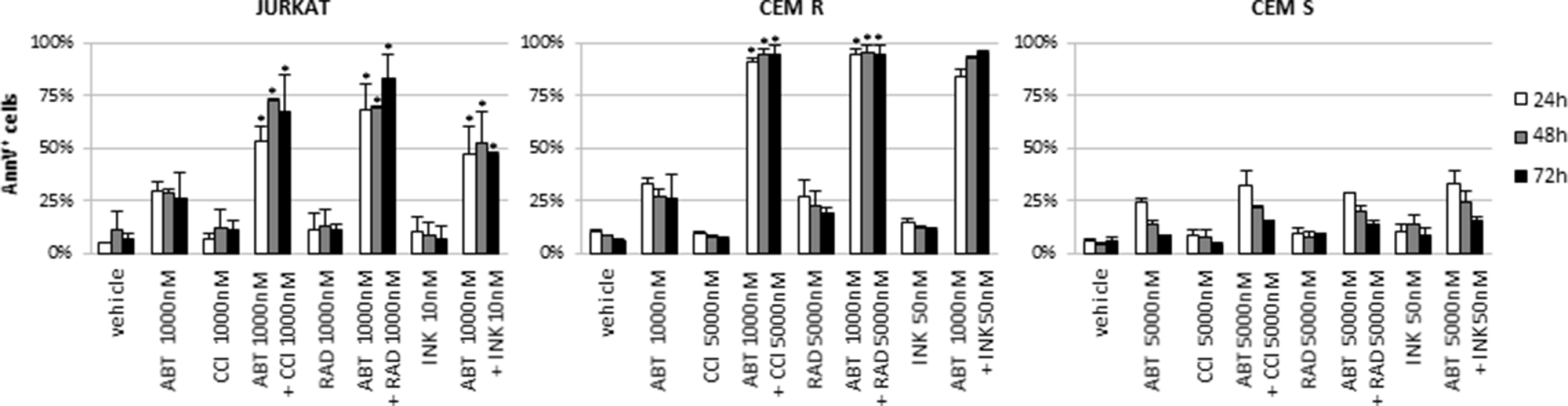
ABT-737 plus RAD001 or INK128 combination confirmed the effectiveness of Bcl-2/mTOR inhibition in ABT-737 resistant ALL cell lines JURKAT, CEM R and CEM S were exposed up to 72 h to ABT-737, CCI-779, RAD001 and INK128, alone or in combination. The concentrations were chosen based on previous studies demonstrating the response of ALL cell lines to these inhibitors. Apoptosis induction was assessed by AnnV/PI staining. Results are shown as mean ± SD of three independent experiments. * for *p* < 0.05 relative to control conditions.

The existence of ABT-737 resistant models led us to investigate whether combined treatment might enhance apoptosis sensitivity. We explored a novel therapeutic approach based on the simultaneous inhibition of the Bcl-2 family proteins and the mTOR pathway activity with ABT-737 plus CCI-779. CCI-779 concentrations were chosen based on previous studies demonstrating the response of ALL cell lines to this inhibitor (data not shown). As shown in Fig. 3, neither ABT-737 nor CCI-779 as single agent induced significant anti-proliferative or cytotoxic effects on the JURKAT, CEM R and CEM S cell lines. By contrast, the combination of ABT-737 and CCI-779 exhibited a synergistic activity (CI < 0.2) on the JURKAT and CEM R cell lines, causing a marked cell growth inhibition and a significant (*p* < 0.01) apoptosis induction. However, combined treatment did not affect the viability and apoptosis rates of the CEM S cell line (Fig. 3). Similar results were obtained with two additional mTOR inhibitors (RAD001 and INK128) (Fig. 4), thus confirming that there were not off-target effects implicated in our findings.

**Figure 5:**
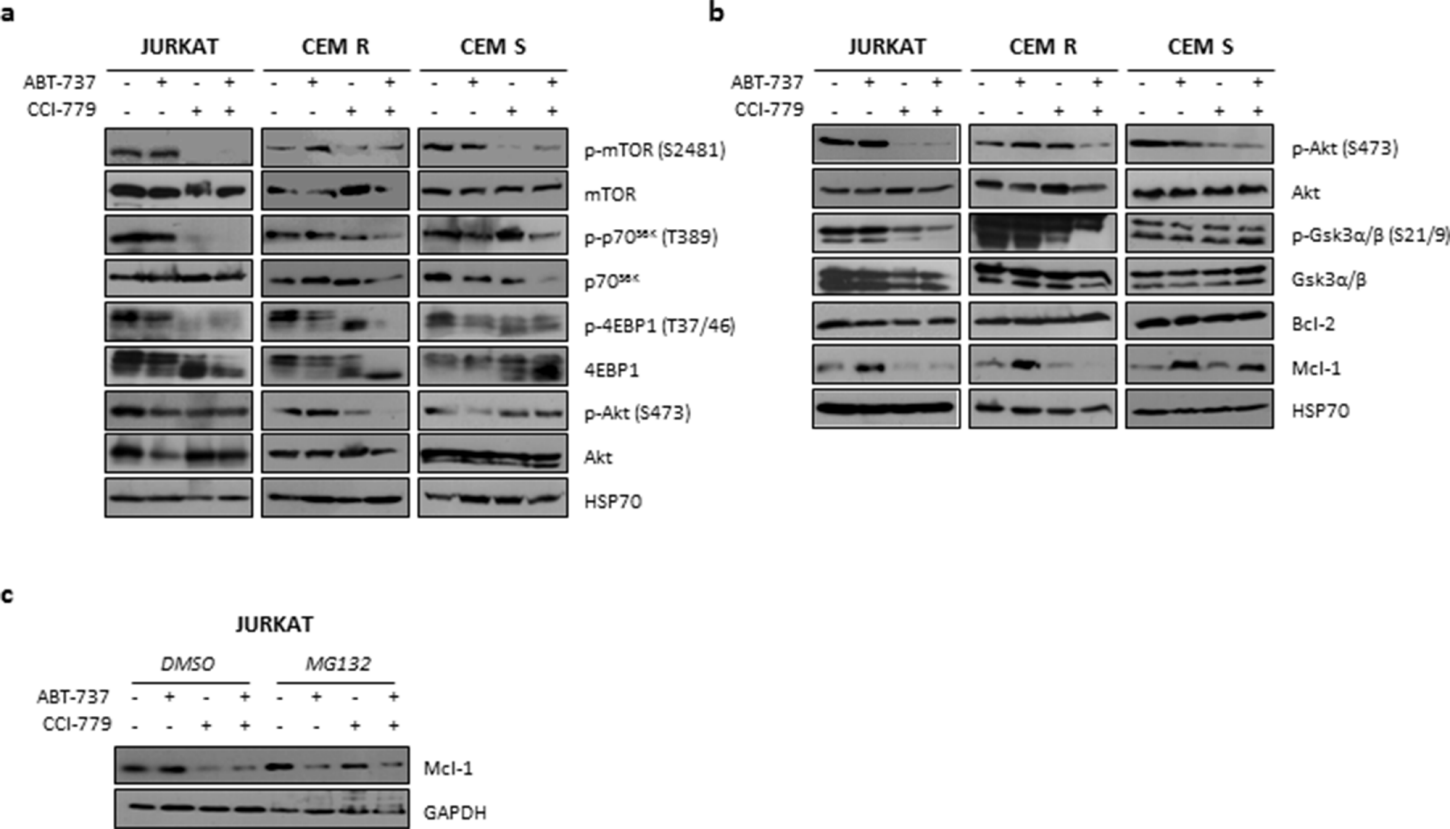
Effects of ABT-737/CCI-779 combination on Bcl-2 and PI3K/Akt/mTOR pathway in ALL cell lines ABT-737 resistant cell lines were cultured in the presence of ABT-737 (1000 nM for JURKAT and CEM R, 5000 nM for CEM S), CCI-779 (1000 nM for JURKAT, 5000 nM for CEM R and CEM S) or their combination and the expression of selected PI3K/Akt/mTOR pathway components and Bcl-2 family members was analyzed by western blot after 3 h **a.** and 24 h **b.** of exposure. Representative immunoblots are shown. **c.** JURKAT cell line was incubated with or without 3 μM MG132 and then exposed to ABT-737 (1000 nM), CCI-779 (1000 nM) and their combination. Mcl-1 expression was assessed at 16 h by western blot analysis. Results are representative of three independent experiments. HSP70 and GADPH were used as loading control.

To understand the molecular mechanisms underlying the synergistic interaction, we incubated JURKAT, CEM R and CEM S cells with ABT-737 and CCI-779, alone and in combination, and protein lysates were obtained at 3 and 24 h. Western blot analysis revealed that a 3 h CCI-779 exposure, alone and in combination with ABT-737, induced the downregulation of the mTORC1 downstream targets p-p70^S6K^(T389) and p-4EBP1(T37/46) in JURKAT and CEM R cells, while no changes were observed in the CEM S cell line (Fig. 5a). Moreover, a prolonged exposure (24 h) to the inhibitors demonstrated that CCI-779, when used in combination with ABT-737, enhanced the dephosphorylation of Akt(S473) and consequently of Gsk3α/β(S21/9) in comparison to single treatment, strongly decreasing Mcl-1 expression in the JURKAT and CEM R cell lines. The combination failed to reduce Mcl-1 levels in the CEM S cell line (Fig. 5b).

### Proteasome is not involved in decreasing Mcl-1 expression

**Figure 6:**
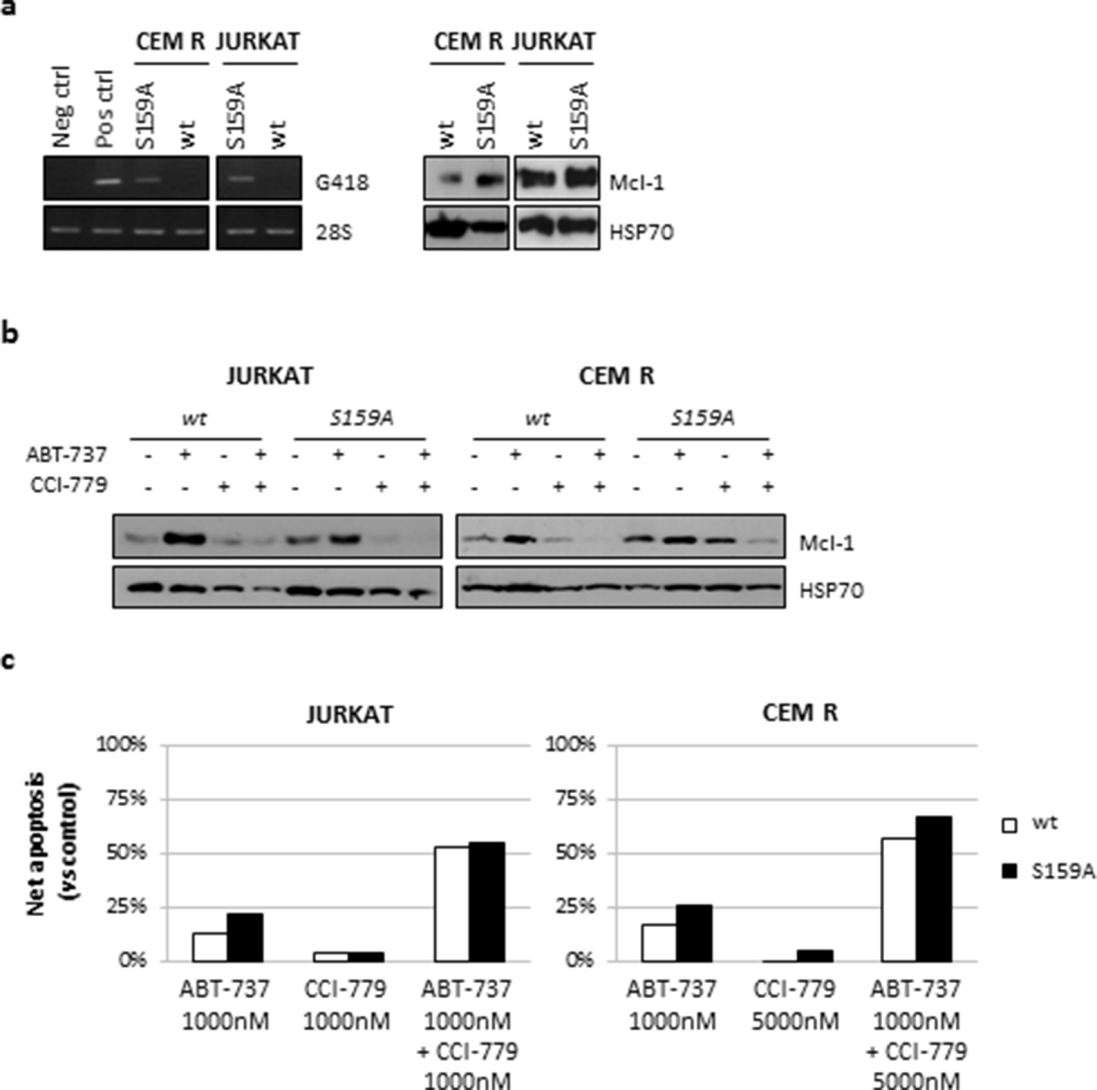
Mcl-1 downregulation is not dependent on proteasomal activity JURKAT and CEM R cell lines were transfected with the Mcl-1 mutant S159A (Mcl-1^S159A^) and the effects on Mcl-1 protein expression and apoptosis induction were analyzed by western blot and AnnV/PI staining, respectively. **a.** Representative RT-PCR (left panels) and western blot analysis (right panels) after transfection in JURKAT and CEM R cell lines. Expression of 28S and HSP70 were used as internal controls. **b.** CEM R cell line was treated with vehicle (DMSO) or ABT-737 (1000 nM) and CCI-779 (5000 nM), alone and in combination. Mcl-1 protein expression was analyzed by western blot after 24 h of exposure. Results from one of at least three independent experiments are shown. HSP70 served as loading control. **c.** Analysis of drug-induced apoptosis in wt and Mcl-1^S159A^-transfected JURKAT and CEM R cell lines after exposure to the indicated concentrations. No statistically significant differences were found between wt and Mcl-1^S159A^-transfected JURKAT and CEM R cells after treatment with ABT-737 as single agent or in combination.

To investigate whether Mcl-1 downregulation would be dependent on Gsk3-induced proteasomal degradation, we treated the JURKAT and CEM R cell lines with the proteasome inhibitor MG132 prior to the addition of ABT-737 and CCI-779 and the expression of Mcl-1 was monitored at 16 h. No increase in Mcl-1 protein level was observed in MG132-treated cells (Fig. 5c), thus indicating that proteasome is not involved in Mcl-1 degradation. To further support that Gsk3-induced Mcl-1 degradation is not involved in ABT-737 resistance, we transfected JURKAT and CEM R cells with a Mcl-1 protein mutant lacking the Gsk3 phosphorylation site S159A (Mcl-1^S159A^) which is known to regulate Mcl-1 stability. An efficient transfection was achieved (Fig. 6a) and both cell lines, as expected, showed a reduction in Mcl-1 downregulation after treatment with ABT-737 and CCI-779, both alone and in combination (Fig. 6b). Unexpectedly, however, cytotoxicity induced by single or dual treatment was not reduced in Mcl-1^S159A^ cells compared to control cell lines (Fig. 6c). Therefore, we hypothesized that inhibition of 4EBP1 and subsequently of protein translation in the JURKAT and CEM R cell lines led to the downregulation of Mcl-1 expression explaining the ability of the ABT-737/CCI-779 combination to trigger apoptosis in JURKAT and CEM R cells but not in the CEM S cell line (Fig. 5b).

### Combined treatment failed to show synergistic activity where Mcl-1 is not the main resistant factor

**Figure 7:**
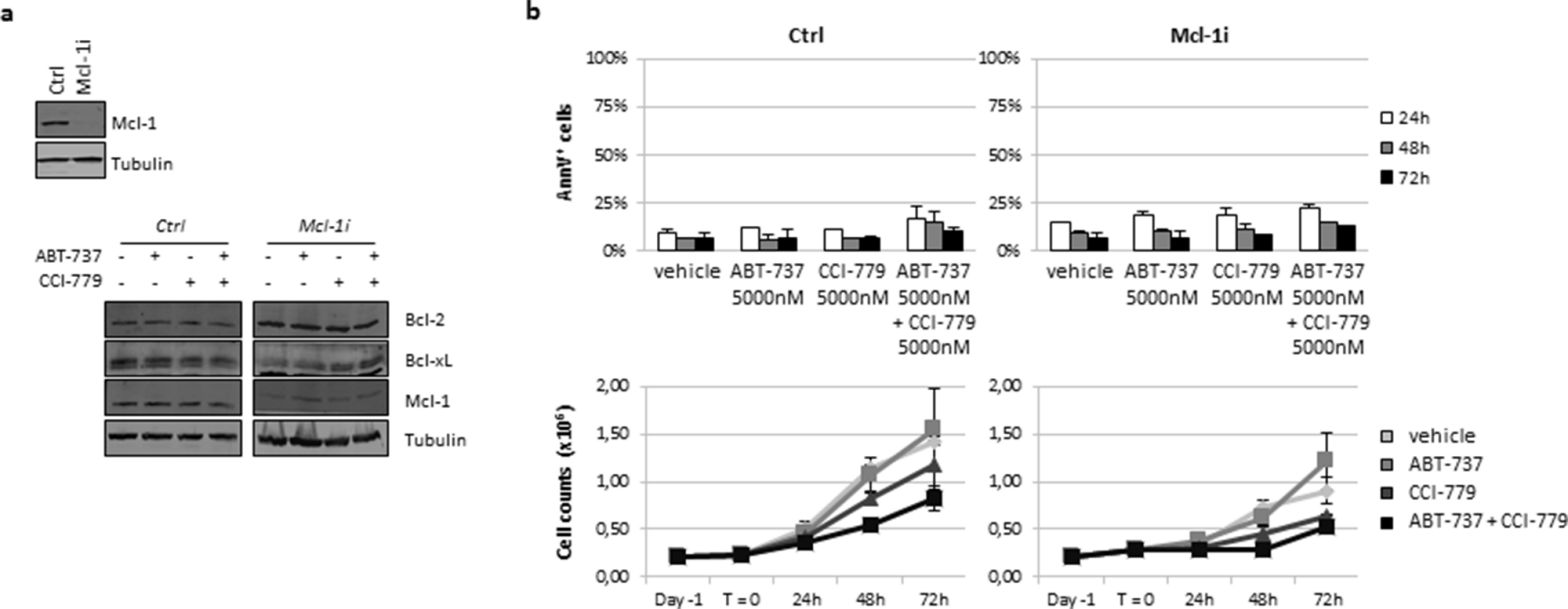
Knockdown of Mcl-1 expression in CEM S cell line failed to enhance drug-induced apoptosis **a.** Analysis of Mcl-1 level (upper panel) and of modulation of Bcl-2 family members protein expression (lower panel) in wt or Mcl-1i CEM S cells prior and after exposure to ABT-737 (5000 nM) and CCI-779 (5000 nM), alone or in combination. Representative immunoblots from one of three independent experiments are shown. Tubulin was used as loading control. **b.** CEM S cell line was treated up to 72 h with vehicle (DMSO) or ABT-737 and CCI-779, alone or in combination. The effects on cell growth (lower panels) and apoptosis induction (upper panels) were analyzed at the indicated times by Trypan blue exclusion method and AnnV/PI staining, respectively. Results are presented as mean ± SD of at least three different experiments. No statistically significant differences were found between wt and Mcl-1i CEM S cells after treatment with ABT-737 as single agent or in combination.

Since the combination of ABT-737 and CCI-779 resulted unable to reduce Mcl-1 expression in the CEM S cell line, we performed RNA interference of Mcl-1 (Mcl-1i). Efficient silencing was achieved without any changes in Bcl-2 and Bcl-xL protein levels (Fig. 7a). We then exposed wt and Mcl-1i cells to ABT-737 and CCI-779, alone and in combination, demonstrating neither changes in protein expression nor increase of apoptosis in Mcl-1i CEM S cells when compared to their controls (Fig. 7b). Only a slight reduction in cell growth was noticed. We thus concluded that in this model Mcl-1 is not the unique determinant of ABT-737 resistance.

### ABT-737 synergized with CCI-779 enhancing apoptosis in resistant ALL primary samples

**Figure 8:**
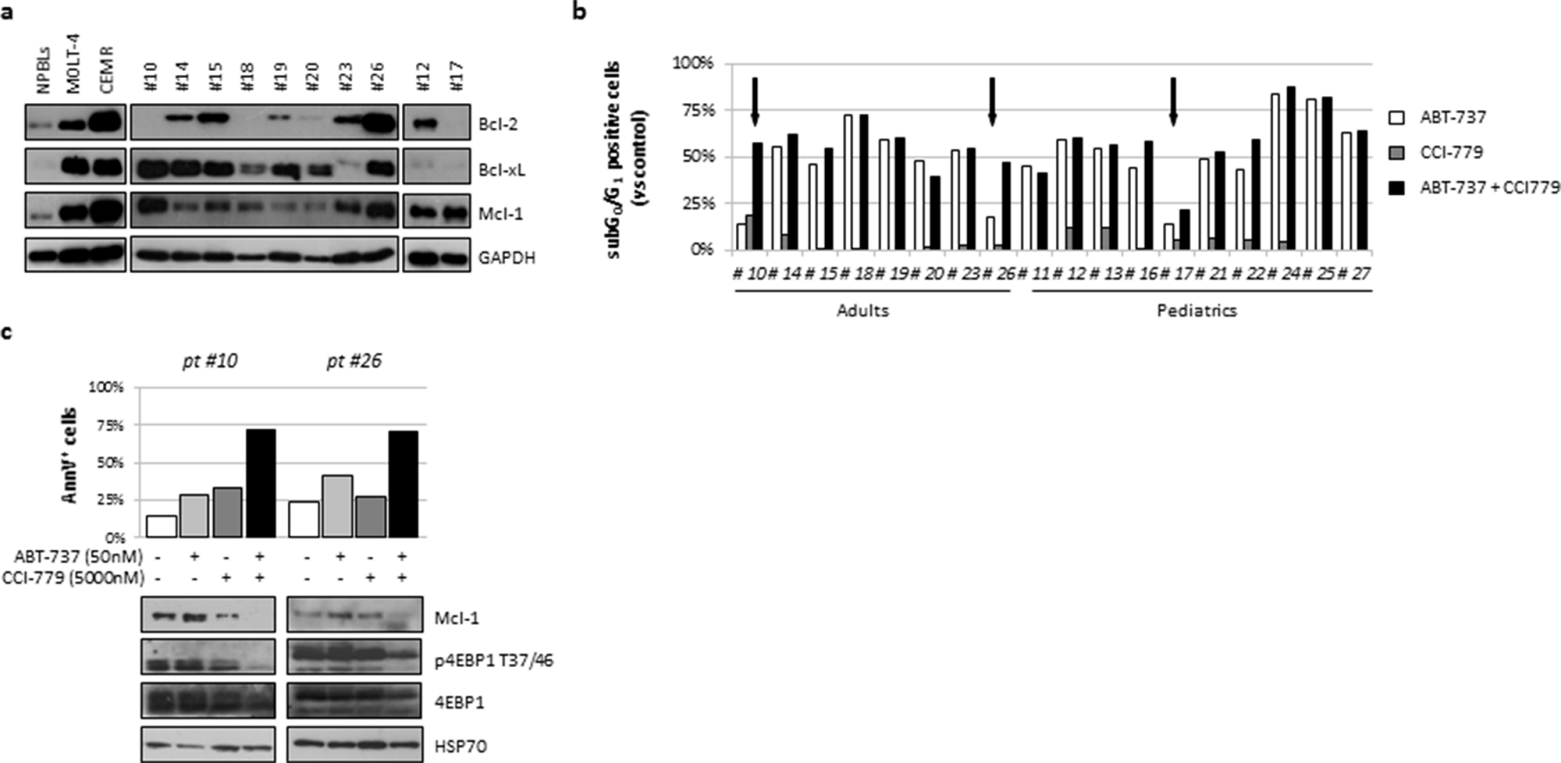
ABT-737 plus CCI-779 combination triggered apoptosis in resistant ALL primary cells **a.** Analysis of basal expression of Bcl-2, Bcl-xL and Mcl-1 in 10 evaluable ALL primary samples. NPBLs, MOLT-4 and CEM R cell lines were used as references. GAPDH served as loading control. **b.** Freshly isolated leukemic blasts from 18 ALL patients were seeded at a density of 1 × 10^6^/mL and treated for 24 h with ABT-737 (50 nM) and CCI-779 (5000 nM), alone or in combination. Apoptosis induction was determined by AO technique. Results are reported as percentage of subG_0_/G_1_ positive cells for individual patient. Synergistic or additive interaction were indicated by black arrows. **c.** Leukemic blasts from sample #10 and #26 were exposed for 24 h to ABT-737, CCI-779 or their combination. Apoptosis induction and protein expression were assessed by AnnV/PI staining and western blot analysis, respectively.

To evaluate the effectiveness of ABT-737 and CCI-779 as a potential therapeutic strategy in ALL, 18 additional primary samples were exposed to the drugs, alone and in combination. As before, basal expression levels of Bcl-2 family members was analyzed by western blot on 10 evaluable samples. Bcl-2 and Bcl-xL were heterogeneously expressed in the majority of samples (7/10 and 9/10, respectively) and almost all samples (9/10) shared the overexpression of Mcl-1 when compared to NPBLs (Fig. 8a). CCI-779 treatment as a single agent demonstrated a modest apoptosis activity (increase of subG_0_/G_1_ peak from 19.9 ± 11.6% (vehicle) to 23.8 ± 15.6% at 5000 nM). By contrast, ABT-737 confirmed its cytotoxic effects also on these samples, inducing a significant (*p* < 0.001) apoptosis induction from 19.9% ± 11.6 (vehicle) to 69.9% ± 21.0 at 50 nM. Importantly, ABT-737 activity was observed in the majority of samples (15/18) while the remaining 3 specimens exhibited less than 20% of net apoptosis (Fig. 8b). Nevertheless, combined treatment was able to induce synergistic (pt #10, #26) or additive (pt #17) pro-apoptotic effects on these 3 samples (Fig. 8b, arrows). No significant differences in sensitivity to ABT-737 as single agent or in combination with CCI-779 were observed when data were sorted by age and/or WBC (data not shown). Further analysis of correlation between ABT-737/CCI-779 sensitivity and protein expression in these three resistant samples revealed that pt #17, which displayed additive effects in combination, was characterized by negligible levels of Bcl-2 and Bcl-xL with high expression of Mcl-1, thus supporting the lower ABT-737 activity as single agent and in combination. Conversely, pt #10 and #26, which displayed elevated protein levels of Bcl-2, Bcl-xL and Mcl-1, showed a synergistic apoptosis induction associated with a marked downregulation of p-4EBP1(T37/46) and Mcl-1 (Fig. 8c), thus resembling the molecular changes previously observed in the JURKAT and CEM R cell lines.

## DISCUSSION

Over the last years, attention in ALL has been focused on the molecular mechanisms underlying drug resistance, developing novel strategies to modulate aberrant signaling rising from acquired lesions [53]. Among these, therapeutic targeting of the deregulated apoptotic machinery represents an attractive area of investigation [54, 55]. Anti-apoptotic Bcl-2 related proteins allow leukemic survival and cell proliferation by interfering with the intrinsic pathway of apoptosis. Thus, inhibition of Bcl-2 family members represents a highly potent stimulus to trigger apoptosis in leukemic cells. ABT-737 is a small-molecule inhibitor of Bcl-2, Bcl-xL and Bcl-w that has been investigated in several human cancers, including hematological neoplasias. Here we first evaluated the activity of ABT-737 as single agent on ALL cell lines and primary samples, assessing the correlation between Bcl-2 family member expression and ABT-737 sensitivity. Previous reports showed that high Bcl-2 or low Mcl-1 expression correlate with *in vitro* sensitivity of several cancer cell lines to ABT-737 [24, 27, 56]. Consistently, Bcl-2 protein expression was associated with drug efficacy in acute myeloid leukemia (AML) [25]. However, in ALL, Bcl-2 dependence rather than basal expression seemed to have a greater impact on cellular response [14]. Indeed, Kang and colleagues [57] showed that levels of anti-apoptotic Bcl-2 family members are not associated with ALL susceptibility to ABT-737. More recently, it has been demonstrated that Mcl-1, Noxa and Bim protein levels significantly impact on *in vitro* resistance of ALL cell lines. By contrast, only Bim rather than Bax, Bak or increased Mcl-1 expression determined *in vivo* sensitivity to ABT-737 in a panel of ALL xenografts from pediatric patients [23]. Therefore, ABT-737 sensitivity in ALL seems to depend on several factors. We found that Mcl-1/Bcl-2 plus Bcl-xL expression ratios determined ALL cell lines *in vitro* response to ABT-737. Indeed, our sensitive cell models were characterized by lower Mcl-1 and upper Bcl-2 plus Bcl-xL protein expression than resistant cell lines. However, it is likely that different factors (e.g. Bim) other than the proteins assessed in this study may affect ABT-737 *in vitro* responsiveness. In agreement with published data [57, 58], we demonstrated that increasing concentrations of ABT-737 further decreased Bcl-xL and Mcl-1 expression only in MOLT-4, a sensitive cell line, inducing significant cell growth reduction and apoptosis at nanomolar concentration. By contrast, protein levels were not affected in CEM S cells, a resistant cell model, showing no significant changes of apoptosis induction even at micromolar doses. Cytotoxic efficacy of ABT-737 was then confirmed on ALL primary samples, with the majority of specimens demonstrating significant apoptosis induction when compared to their controls. By contrast, normal CD34^+^ cells were remarkably resistant to ABT-737, as already shown by Konopleva *et al*. [25],

Interestingly, we found a resistant primary sample in which Mcl-1 expression was not affected by ABT-737 exposure. Conversely, analysis of two sensitive ALL samples revealed a marked Mcl-1 downregulation following ABT-737 exposure, likely due to apoptosis induction. These results, in accordance to published data [56], support the contribution of Mcl-1 downregulation in the ABT-737-induced cytotoxicity.

To overcome BH3 mimetics resistance, we explored a novel therapeutic approach by using the mTOR inhibitor CCI-779 in ALL cell lines and primary samples. The PI3K/mTOR pathway is frequently found constitutively activated in ALL [35, 36, 59] and targeted inhibition of mTOR, alone or in association with PI3K, has been investigated at multiple levels, generally displaying better results *in vitro* than *in vivo*. Recently, it has been demonstrated that activated Akt may decrease ABT-737 activity, suggesting a functional role of this signaling also in protecting leukemia cells from BH3 mimetic-induced apoptosis [60]. Consistently, simultaneous inhibition of the PI3K/mTOR pathway, by BEZ235, and Bcl-2/Bcl-xL, by ABT-737, strongly potentiated cytotoxicity of single agents and markedly reduced colony formation in multiple AML cell lines and primary blast specimens while exerted only modest toxicity toward normal hematopoietic progenitors [60]. Moreover, Jin and colleagues [61] showed that the combination of GDC-0941 and ABT-737 triggered mitochondrial apoptosis in AML cells co-cultured with stromal cells under hypoxic conditions, thus suggesting the efficacy of this approach even under the protective conditions afforded by the bone marrow microenvironment. It has also been demonstrated that PI3K/mTOR signaling contributed to ABT-737 resistance in Burkitt lymphoma cells, and their inhibition through dual or active site mTOR inhibitors induced synergistic caspase activation, rendering chemoresistant cells sensitive to apoptosis induction [62]. In addition, data obtained on different solid tumors [31, 63, 64] showed that blockade of mTOR signaling and Bcl-2 activity triggered massive *in vitro* apoptosis by loss of mitochondrial membrane potential, activation of caspases and Mcl-1 downregulation and was highly synergistic *in vivo* providing tumor regressions without notable hematologic suppression. Since numerous of the aforementioned inhibitors have shown promising results in different stages of clinical trials, alone or in combination with chemotherapy [clinicaltrials.gov], it has been proposed that the simultaneous inhibition of the mTOR pathway and Bcl-2 activity in cancer cells, even in those resistant to single agents, may have important clinical implications. We found that CCI-779, RAD001 and INK128, as single agents, modestly impaired resistant cell line proliferation, resulting in a no significant pro-apoptotic activity. By contrast, co-targeting of Bcl-2 and mTOR activity with all the above inhibitors strongly sensitized resistant JURKAT and CEM R cell lines to apoptosis, thus reverting the acquired ABT-737 resistance. Interestingly, since CEM R cells are known to be insensitive to vinblastine [65], we hypothesized that combined treatment can be effective also in other chemoresistant models even those MDR1-related. However, the CEM S cell line failed to show any apoptotic increase after exposure to all tested combination, suggesting an analysis of protein expression to better understand the biological reasons underlying synergistic interaction between the aforementioned drugs. We observed that CCI-779, especially when in combination with ABT-737, inhibited the mTORC1 pathway activity by decreasing phosphorylation levels of p70^S6K^ and 4EBP1 only in JURKAT and CEM R, thus reinforcing the idea of a context-dependent role of mTORC1 in the increased ABT-737-mediated cell death [31]. Moreover, after 24 h of treatment, CCI-779 also affected the mTORC2 pathway, as demonstrated by the downregulation of p-Akt(S473) and p-Gsk3α/β(S21/9), indicating that prolonged exposure to rapamycin derivative may be effective, at least in part, also on PI3K downstream targets. Of note, combined treatment markedly decreased Mcl-1 protein levels in the JURKAT and CEM R cell lines, while not affecting its expression in CEM S cells. We excluded the involvement of proteasomal degradation in ABT-737-induced Mcl-1 downregulation since protein levels appeared not increased in cells treated with MG132, a well-known proteasome inhibitor, compared to their controls. We also transfected a mutant of Mcl-1 which lacks the S159 residue, a known Gsk3 phosphorylation site that marks Mcl-1 for proteasomal degradation, in the JURKAT and CEM R cell lines. Although an efficient transfection was achieved and both cell lines showed a reduction in Mcl-1 downregulation, we unexpectedly detected no reduction in apoptosis induction after single or combined treatment, further supporting the idea that proteasomal degradation is not involved in Mcl-1 degradation. These finding led us to hypothesize that inhibition of 4EBP1-dependent protein translation may be important for the synergistic interaction between ABT-737 and CCI-779. Further experiments are needed to clarify unequivocally the precise contribution of molecular mechanisms (e.g. caspase-dependent cleavage) in determining Mcl-1 levels on ALL cells.

However, our results demonstrated that in some model, as the CEM S cell line, Mcl-1 is not a unique determinant of ABT-737 resistance. Indeed, when we knocked down protein expression by RNA interference, we observed no changes in treatment cytotoxicity, even after exposure to the combination. Different groups have already reported that, in certain contexts, Mcl-1 downregulation although required is incapable itself of initiating apoptosis [66, 67]. In addition, loss of Bim, combined absence of Bax and Bak or overexpression of Bcl-xL and Bcl-w have been shown to make lymphoma cells resistant to ABT-737 *in vitro* and *in vivo*, even in the absence of endogenous Mcl-1 [68], thus indicating that targeting of more than one antiapoptotic protein may be required for the full engagement of the apoptotic machinery. Therefore, we concluded that Mcl-1 downregulation likely cooperates with other independent mechanisms to support ABT-737 resistance in ALL.

The effectiveness of the ABT-737 and CCI-779 combination as a novel therapeutic strategy was further evaluated on additional primary ALL samples. CCI-779 treatment as monotherapy demonstrated only a modest apoptosis activity on these samples, whereas ABT-737 confirmed its cytotoxic effects inducing significant apoptosis increase on the large majority of samples. Importantly, combined treatment was able to sensitize ABT-737 resistant samples to apoptosis in an additive or synergistic fashion, thus overcoming the acquired resistance to ABT-737. Of note, analysis of protein expression revealed that samples that displayed additive effects in combination were characterized by negligible levels of Bcl-2 and Bcl-xL with high expression of Mcl-1, supporting lower drug activities. Conversely, the other ABT-737 resistant samples displayed elevated protein levels of Bcl-2, Bcl-xL and Mcl-1, showing a synergistic apoptosis induction after combined treatment associated with a marked downregulation of Mcl-1. However we cannot exclude the existence of primary samples that exhibit a resistance phenotype as the CEM S cell line (i.e. not dependent only from Mcl-1).

In conclusion, our study show the efficacy of BH3 mimetics as single agent in the majority of the ALL samples and demonstrate that Mcl-1 represents the main resistance factor to the BH3 mimetic ABT-737 in ALL cell lines and primary samples. Co-targeting of the Bcl-2 family proteins and mTOR pathway effectively enhanced single drug cytotoxicity by downregulating mTORC1 activity and Mcl-1 expression. Additional resistant mechanisms, however, should be taken into account since, in certain ALL models, Mcl-1 modulation failed to overcome by itself ABT-737 resistance, thus suggesting the existence of other mechanisms of resistance to BH3 mimetics.

## MATERIALS AND METHODS

### Primary samples, cells cultures and *in vitro* treatments

Bone marrow or peripheral blood primary samples from 14 adult and 13 pediatric ALL patients (Table 1) were collected after informed consent in accordance with the Helsinki Declaration and approved by the Institutional Review Board of the Sapienza University of Rome and normal peripheral blood lymphocytes (NPBLs) were purified by Ficoll-Hypaque (Sigma-Aldrich, MO, USA) density-gradient centrifugation. Only specimens with blast counts > 70% were included in this study. For *in vitro* treatments, ABT-737 (kindly provided by Abbott Laboratories) and CCI-779 (Novartis, Basel, Switzerland) were prepared in DMSO and ethanol to a stock concentration of 10 and 20 mM, respectively. RAD001 (Sigma-Aldrich) and INK128 (Selleckchem, Germany) were both diluted in DMSO to a stock concentration of 10 mM. ALL cell lines (MOLT4, RS4;11, JURKAT, CEM R and CEM S) were cultured in RPMI 1640 medium (Euroclone, Milan, Italy) containing 10% heat inactivated fetal bovine serum, 2 mM l-glutamine and antibiotics at 37°C under 5% CO_2_ - 95% air and harvested in log-phase growth. For each experiment, cell lines and primary samples were plated at concentration of 0.2 × 10^6^/mL and 1 × 10^6^/mL, respectively. Viability was assessed by triplicate Trypan blue exclusion counting. To establish the optimal concentration for drug treatment, MTT [3-4,5-dimethylthythiazol-2-yl)-2,5-diphenyltetrazolium bromide] assays (Sigma-Aldrich) were performed. Cell lines and primary samples were incubated up to 72 h with ABT-737 (ranging from 10 to 5000 nM), CCI-779 (10 to 20000 nM), RAD001 (500 to 10000 nM) and INK128 (1 to 500 nM) alone and in combination. For proteasome inhibition, 3 μM of MG-132 (Sigma-Aldrich) was added to the medium 3 h before drugs administration.

**Table 1:** Clinical and biological features of ALL primary samples included in this study

#	Type of ALL	Sex	Age	WBC (10^3^/ml)	Blasts	Cytogenetics & molecular alterations
1	B-ALL	m	adult	15,4	> 70%	t(9;22)
2	B-ALL	f	adult	15	> 70%	t(9;22)
3	B-ALL common	f	adult	82,7	88%	t(9;22)
4	B-ALL common	m	pediatric	123,5	83%	t(9;22)
5	B-ALL	f	adult	22,4	100%	t(4;11)
6	B-ALL	m	pediatric	69,5	85%	mol neg
7	B-ALL	m	adult	232	92%	t(4;11)
8	B-ALL common	f	adult	41,2	100%	t(9;22), t(q34,p11)
9	B-ALL	m	pediatric	20,7	87%	t(11;19)
10	T-ALL	m	adult	470	92%	46 XY, t(7;11)(q33,p15) [8], idem del6(q13,q21)
11	B-ALL common	f	pediatric	26,1	80%	mol neg
12	B-ALL common	f	pediatric	7	> 70%	49 XXX, +8, +21 [2]/46 XX [19], t(12;21)
13	B-ALL common	f	pediatric	42,8	88%	mol neg
14	T-ALL	m	adult	28	70%	46 XY, mol neg
15	Lymphoma leuk	m	adult	92	80%	mol neg
16	B-ALL common	f	pediatric	3,7	> 70%	mol neg
17	mature B-ALL	f	pediatric	13,1	86%	t(8;14)
18	T-ALL	m	adult	22	80%	mol neg
19	B-ALL	m	adult	9	96%	mol neg
20	B-ALL	f	adult	321	97%	t(9;22)
21	B-ALL common	m	pediatric	173,1	91%	na
22	B-ALL common	m	pediatric	103	94%	t(12;21)
23	Lymphoma leuk	m	adult	55,4	84%	t(11;19)(q32,q13)
24	T-ALL	m	pediatric	361,5	98%	na
25	B-ALL common	m	pediatric	99,1	88%	na
26	B-ALL	f	adult	2,2	90%	na
27	B-ALL	m	pediatric	345,4	98%	t(4;11)

Abbreviations: mol neg, molecular negative; na, not assessed

### Apoptosis and cell-cycle analysis

Cell cycle analysis was performed by Acridine Orange technique, as previously described [69]. To evaluate apoptosis levels, cells were examined over a culture period of 24–72 h using Annexin V-fluorescein isothiocyanate (FITC)/propidium iodide (PI) staining, as previously described [70]. Apoptosis induction was further measured by evaluating the subG_0_/G_1_ peak using AO technique, as described [71]. Flow cytometric analyses were carried out using both FACSCan and Accuri C6 flow cytometer (BD Biosciences, CA, USA). Cytotoxic activity induced by *in vitro* treatments was evaluate by measuring the net apoptosis rate obtained from the difference between subG_0_/G_1_ value of each condition and of that of controls.

### Western blot analysis

Protein expression of Bcl-2 family and PI3K/Akt/mTOR pathway key components of ALL cell lines and primary samples with available amount of cells was analyzed by western blotting. Cells were exposed to ABT-737 and CCI-779 and lysed in ice-solution containing 10 mM NaF, 1 mM Na_3_VO_4_, 150 mM NaCl, 1 mM MgCl_2_, 1 mM CaCl_2_, 0.1% NaN_3_, 10 mM iodoacetamide, 3 mM PMSF and 1% Triton X-100 (Sigma-Aldrich) supplemented with protease inhibitor cocktail (Roche Diagnostics, Indianapolis, USA). Equal amounts of proteins were subjected to SDS–PAGE. After electrotransfer on nitrocellulose (Bio-Rad Laboratories, CA, USA), membranes were blocked in 5% w/v non-fat dry milk and incubated overnight with primary antibodies, according to manufacturer's instruction. Blots were then probed with HRP-conjugated secondary antibodies for 1 h at room temperature and developed using ECL reagent. Resulting signals were collected on x-ray films, digitally scanned and quantified using Image J software (NIH, Bethesda, MD, USA).

The antibodies used for Western blot were as follows: anti Bcl-2 (DAKO Corp, Carpinteria, CA, USA), anti Mcl-1 (Santa Cruz Biotechnology, CA, USA), all the others antibodies were purchased from Cell Signaling Technology (Beverly, Massachusetts, USA).

### Transfection

Electroporation was performed by Bio-Rad Gen-Pulser II apparatus (Bio-Rad). JURKAT cells were counted, centrifuged and resuspended in PBS (Euroclone). Four hundred microliters of cell suspension (6 × 10^6^ cells) was mixed with 20 μg pcDNA3.1-hMcl-1(S159A) (Addgene plasmid 25374). Cells were electroporated in a 4-mm cuvette at 310V, 975μF. CEM R cells were counted, centrifuged and resuspended in PBS. Four hundred microliters of cell suspension (4 × 10^6^ cells) was mixed with 20 μg of pcDNA3.1-hMcl-1(S159A). Cells were electroporated in a 4-mm cuvette (Bio-Rad) at 250 V, 950 μF. Cells were stored on ice for 10 min and then gently transferred to 15 ml of culture media. Stable transfectants were selected in medium containing G418 (Invitrogen, Milan, Italy) for fifteen days.

### Plasmids and Infection

Each lentiviral shRNA construct, previously described [25], was transfected into 293T cells using Lipofectamine 2000 (Invitrogen). Following 72 h post-transfection, virus-containing supernatants were collected and used to infect cells by the spin-inoculation method. Briefly, 2 × 10^6^ cells were added with 2 ml of virus and 8 μg/mL polybrene, transferred in a multiwell plate and then centrifuged at 1500 g for 90 minutes at 30°C. Once the cells have entered log phase, selection was applied to obtain stable clones.

### Statistical analysis

All data were obtained from at least three independent experiments and are presented as means ± SD (standard deviation). Significant differences between experimental values was determined by using the Student's *t* test. For combination experiments, analysis of synergism was assessed by using the Chou-Talalay method and the CalcuSyn software (Biosoft, Cambridge, MA, USA). CI values lower than 0.8 were considered synergistic, between 0.8 and 1.2 additive, and greater than 1.2 antagonistic.
